# Mental wellbeing in doctors: the measure matters! development of a core outcome set for measuring wellbeing in doctors

**DOI:** 10.1192/bjo.2021.630

**Published:** 2021-06-18

**Authors:** David Baldwin, Aimee O'Neill, Julia Sinclair, Gemma Simons

**Affiliations:** 1 Professor of Psychiatry and Head of Mental Health Group, University of Southampton Faculty of Medicine Honorary Professor of Psychiatry, University of Cape Town, South Africa Visiting Professor, Shandong Mental Health Centre, China; 2 Centre for Workforce Wellbeing, University of southampton

## Abstract

**Aims:**

To achieve a consensus Core Outcome Set for measuring mental wellbeing in doctors.

Hypothesis: A minimum set of valid, reliable and practical wellbeing measures is needed for doctors.

**Background:**

The importance of doctors’ mental wellbeing to everyone using Health Care is highlighted by the levels of burnout reported in doctors around the world. In 2019 a number of UK policy documents made recommendations for the wellbeing of doctors, but how those wellbeing interventions are evaluated needs to be defined. Core Outcome Sets are increasingly being used in medicine to prevent waste in research, by recommending the inclusion of a minimum set of valid, reliable and practical measures. An operational definition and Core Outcome Set for wellbeing in doctors is needed to meaningfully progress the work in this field.

**Method:**

The Centre for Workforce Wellbeing (C4WW), a collaboration between the University of Southampton and Health Education England, was created to support research into the nature, assessment and enhancement of wellbeing in physicians. A Systematic Review of wellbeing measures used in doctors and the robustness of those measures, along with surveys of 250 UK doctors of all grades and specialities and patient and public involvement is informing what a core outcome set could be. A Delphi Study among 37 UK experts has been initiated to establish the consensus Core Outcome Set.

**Result:**

Publication of research into doctors’ wellbeing is growing internationally. In the UK alone data are being captured by multiple national organisations including: the Care Quality Commission, General Medical Council, British Medical Association and the Royal Colleges. Health and Social Care Organisations are, therefore, keen to “do something” and are spending money on wellbeing interventions with little, or no, evidence base and a lack of appropriate, comparable evaluation. A Core Outcome Set for measuring wellbeing in doctors is ethically required to reduce waste, to replace burnout measures and to refine wellbeing interventions.

**Conclusion:**

Wellbeing measures that actually measure wellbeing, and not burnout, which are validated, reliable and practical, are needed to inform local organisational, national government and international research policy. An absence of burnout does not equate to wellbeing. The focus of measurement needs to shift to capture in what contexts we thrive, not just survive. If everyone used the same Core Outcome Set to measure mental wellbeing, direct comparisons could be made, and money invested, in creating infrastructure, processes and cultures that really work.

Health Education England funded PhD.

